# The Effect of Water upon Deep Eutectic Solvent Nanostructure: An Unusual Transition from Ionic Mixture to Aqueous Solution

**DOI:** 10.1002/anie.201702486

**Published:** 2017-05-29

**Authors:** Oliver S. Hammond, Daniel T. Bowron, Karen J. Edler

**Affiliations:** ^1^ Centre for Sustainable Chemical Technologies University of Bath Claverton Down Bath BA2 7AY UK; ^2^ ISIS Neutron and Muon Source STFC Rutherford Appleton Laboratory, Harwell Oxford OX11 0QX UK

**Keywords:** deep eutectic solvents, ionic liquids, nanostructures, neutron diffraction, sustainable chemistry

## Abstract

The nanostructure of a series of choline chloride/urea/water deep eutectic solvent mixtures was characterized across a wide hydration range by neutron total scattering and empirical potential structure refinement (EPSR). As the structure is significantly altered, even at low hydration levels, reporting the DES water content is important. However, the DES nanostructure is retained to a remarkably high level of water (ca. 42 wt % H_2_O) because of solvophobic sequestration of water into nanostructured domains around cholinium cations. At 51 wt %/83 mol % H_2_O, this segregation becomes unfavorable, and the DES structure is disrupted; instead, water–water and DES–water interactions dominate. At and above this hydration level, the DES–water mixture is best described as an aqueous solution of DES components.

Deep eutectic solvents (DESs) are a compositionally diverse range (>10^6^) of low‐transition‐temperature mixtures, and represent a set of intrinsically “designer solvents”, prepared by mixing hydrogen‐bonding salts and neutral species in the eutectic molar ratio.^1^ The physicochemical properties of DESs are related to those of ionic liquids and their mixtures;^2^ DESs are hybrid systems where molecular ionic clusters are found within a complex and disordered hydrogen‐bonding network.^3^ This nanostructure can be adjusted by selection of the mixing ratio and molecular chemical moieties,^4^ and this additional degree of design freedom has aided the development of DESs as “greener” alternative media for organic and inorganic synthesis,^5^ electrochemistry, separation, extraction, and biotransformations.^6^

DESs are made of coordinating, hydrogen‐bonding ions and molecules, making them strongly water‐miscible and hygroscopic. Latent absorbed water is unavoidable, and impacts upon the physicochemical properties of DESs, such as the melting point, with inadequate characterization leading to poor reproducibility.^7^ A relatively new approach leverages the favorable physicochemical properties of DES/water mixtures, such as lowered viscosity.^8^ Trends in these properties suggest that there is an upper limit to this hydration above which DESs are more like aqueous solutions.^9^, ^10^, ^11^ However, it is not known how far such mixtures can be hydrated before they cease to be DESs on a nanostructural level because only a limited compositional range has been probed experimentally in detail by NMR spectroscopy,^12^ which has also been used extensively for ionic liquid (IL)/water mixtures.^13^, ^14^ The effect of water on DESs, and hence their classification, therefore remains one of the most significant unanswered questions in the field; do they resemble ILs, ionic mixtures, or merely solutions of ions? Herein, we analyzed the nanostructure of the archetypal choline chloride/urea DES^15^ across a wide range of hydration. In doing so, we have identified a structure transition point from a DES/water mixture to a state closer to an aqueous solution of individually solvated DES components. This fundamental insight will aid the understanding, development, and application of DESs as advanced reaction and processing media.

Aqueous mixtures were prepared by mixing DES with water in a series of DES/water molar ratios (*x*), referred to as reline‐*xw*, e.g., 1:2:5 choline chloride/urea/water is reline‐5*w*. DES/water molar ratios of 1, 2, 5, 10, 15, 20, and 30*w* were used, and these are given as the corresponding weight and molar percentages in the Supporting Information. The solvent structures were determined by neutron total scattering, with five H/D isotope contrasts per composition. Atomistic models were resolved to the data using empirical potential structure refinement (EPSR).^16^ Details are provided in the Supporting Information.

No small‐angle scattering (*Q*=0.01–0.5 Å^−1^, *d*=1.2–60 nm) was observed, demonstrating that these mixtures are not classically phase‐separated. Therefore, the neutron diffraction data in Figure 1 demonstrate a nanostructure transition on the intermolecular level from that of pure DES to that of water. Pure deuterated reline has two primary scattering features at *Q*=1.45 and 2.15 Å^−1^, and the latter merges with the D_2_O peak found at 2 Å^−1^. The 1.45 Å^−1^ structuring decreases (relative to that at 2 Å^−1^) as the water mole fraction increases. At 10*w* of hydration, the 2 Å^−1^ correlation is slightly greater, yet at ≥15*w*, the interaction at 1.45 Å^−1^ disappears almost completely, leaving one dominant structuring feature. The data therefore highlight a contraction in the major intermolecular interaction length (which is the most likely mean pair separation distance in the liquid) from 4.3 Å in the pure DES to 3.1 Å, the value found for water. This process is gradual up to 10*w*, and sudden at 15*w*. A similar contraction was observed at wider angles, with the pure DES diffraction features (6 Å^−1^; 1.05 Å) converging upon the water peak (8 Å^−1^; 0.79 Å) at 15*w*.

**Figure 1:**
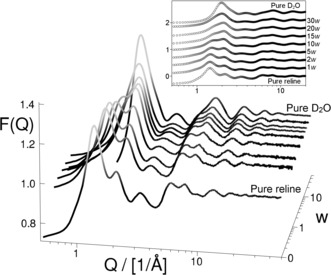
Experimental neutron diffraction data as 3D or 2D (inset) plots for perdeuterated DES mixtures. Data for reline^3^ and D_2_O^17^ are as previously published, with the D_2_O data plotted at *w*=50 for convenience. The ordinate F*(Q)* is normalized to units of barn atom^−1^ steradian^−1^.

The EPSR models equilibrated closely to the neutron data (see the Supporting Information). Atomistic data from EPSR models were interpreted by integrating partial radial distribution functions (pRDFs) up to the first minima (*R*_max_). The resulting coordination number (*N*_coord_) describes the number of nearest‐neighbor molecules, and these are displayed in Figure 2. High *N*_coord_ values indicate important structural features in the disordered liquid. A discontinuity is observed at 1*w*, a concentration chosen to reflect the latent absorbed water in DES.^7^ All DES interactions are weakened upon addition of 1 molar equivalent of water, except for the strengthened choline–urea hydrogen‐bonding interaction (OH⋅⋅⋅NH_2_), which is reflected in the coordination numbers shown in the Supporting Information. This unexpected increase in intermolecular interaction strength explains why DES‐1*w* systems do not undergo the anticipated viscosity reduction.^8^, ^12^ Whilst 80 % (on average) of the original nanostructure is retained at 1*w*, even low‐level DES/water mixtures clearly differ from pure DES, with water contributing to the nanostructure and hence altering the physicochemical properties. As such, appropriate practice is to accurately determine and report the water content of DES.^18^

**Figure 2:**
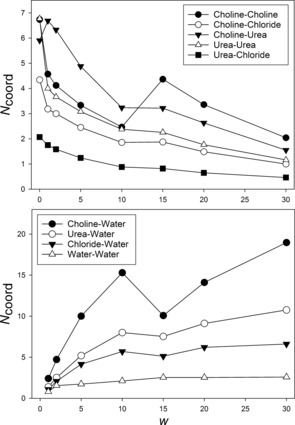
Experimental intermolecular coordination numbers for DES–DES interactions (top) and DES–water interactions (bottom). Reline‐0*w* data are as previously published.^3^

The interactions between the DES components are weakened systematically but non‐linearly as water is introduced, and are retained up to high water mole fractions (10*w*). On average, 50 % of the pure DES nature remains at 10*w* (77 mol % water). Whilst weakening of the “DES–DES” interactions (namely the choline–urea, choline–chloride, choline–choline, urea–chloride, urea–urea, and chloride–chloride hydrogen bonds, which may be strong or weak, and nonionic, ionic, or doubly ionic)^19^ by water is anticipated,^20^ this deviates significantly from Raoult's ideal entropic dissolution.^21^ This is corroborated by the DES/water *N*_coord_ values (namely choline–water, urea–water, and chloride–water interactions), most of which increase as a function of hydration (Figure 2, bottom). A noteworthy exception to this is the choline–water correlation, which increases super‐stoichiometrically, seemingly because of a strong hydration preference relatable to the aqueous solvation of ammonium salts.^22^ Therefore, up to 10*w*, the system resists hydration to retain most of the initial nanostructure. In this solvation regime, water contributes to the slightly ordered hydrogen‐bonding network in the mixture,^19^ and is mostly sequestered by choline cations through short‐range Coulombic and hydrogen‐bonding interactions. This explains the tolerance of the DES nanostructure and properties towards hydration, and can be related to the solvophobic accommodation of solutes in ILs;^2^ “solvent‐separated ion pairs” are seen in many IL/cosolvent mixtures.^21^ A transient water‐rich sequestered domain around choline helps to rationalize the unusual properties of hydrated DESs, such as low water activity^23^ and a high water self‐diffusion coefficient.^12^ The retention of the DES “pseudo‐IL” character at such low ionic strengths is remarkable.

At 15*w* (83 mol %, 51 wt % H_2_O), a second discontinuity in the intermolecular interactions is observed. Our experimentally validated models allow us to assign this as the nanostructure transition from a “water‐in‐DES” to a “DES‐in‐water” regime. The choline–choline and choline–water interactions are most markedly affected. At 15*w*, the number of water molecules solvating choline falls from 15 to 10 whilst the choline–choline *N*_coord_ rises from 2.5 to 4.4. Above this level, DES–DES interactions continue to weaken while the DES–water correlations intensify. Furthermore, except for the choline–choline and water–water interactions, it is surprising not to observe any length scale changes in the DES–DES interactions from this point to 30*w*. At 15*w*, the water–water *N*_coord_ plateaus at a value equal to that of pure water.^24^ Therefore, above this point, it is inappropriate to describe the system as a DES, and it should instead be considered as a solution of DES components in water. Importantly, this nanostructure transition point correlates with trends in the physical properties of cholinium DES/water mixtures.^8^, ^12^, ^23^

Specific nanostructure analysis shows only subtle differences across the hydration range, shown as 3D spatial density function (SDF) plots in Figure 3, which are projections of the most likely configurations. Even at high *w*, the preferred orientations of urea and chloride around choline cations are retained. However, choline–choline structuring is affected by the strong water interaction. Water systematically occupies a radial hydrogen‐bonded solvation band around the choline hydroxy group at shorter distances than urea or chloride, and along the urea hydrogen‐bonding axes. At 15*w*, the choline–urea interaction is diminished because the close‐range choline–water and urea–water interactions dominate. The hydration of the DES components increases with the water volume fraction, and at 15*w*, the urea molecules have a saturated first hydration shell, with a similar increase in crowding for choline. Additional SDF plots of water–chloride (and choline–water at 10*w*, the maximized interaction point) are given as Supporting Information. These demonstrate that the solvation of water by chloride increases with hydration, further signifying the transition from a DES to an aqueous solution. Breakdown of DES structure therefore correlates with the point where DES–water interactions dominate over DES–DES interactions.

**Figure 3:**
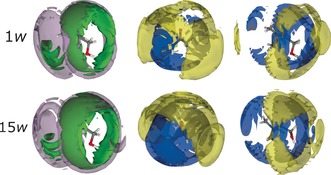
SDF plots describing the 3D nanostructure of reline‐1*w* (top) and ‐15*w* (bottom). Isosurfaces denote chloride (green), urea (lilac), choline (yellow), and water (blue) molecules at the 7.5 % probability level. The central molecules are choline (left), urea (center), and choline (right).

From this insight, we propose a mechanism for the transition from hydrated DES to a DES aqueous solution. Between 1*w* and 15*w*, there is a solvent‐separated ionic cluster regime, with preferential water–choline nanostructuring driven by solvophobic segregation. This explains the tolerance of the DES towards hydration and trends in the physicochemical properties.^2^ However, this sequestered water reaches an overcrowding point (15*w*). Here, it becomes preferable for the DES components to be fully solvated by water and for the system to become an aqueous solution. Some DES–DES bonding still exists in this regime because the DES components are not non‐interacting, ideal solutes. However, the proportion of these interactions relative to the water–water pRDFs is so low that such systems do not represent the DES nanostructure, and should not be characterized as DESs.

In summary, we have analyzed the nanostructure of the hydrated reline DES system across a wide hydration range. At low levels (≤1*w*), water contributes slightly to (rather than disrupting) the hydrogen‐bonding network, and strengthens choline–urea bonding. This alters the structure enough that it is important for the water content of DES to be characterized. Between 2*w* and 10*w*, the DES–water mixture is in a regime where DES clusters still exist, but are separated by the diluent. DES intermolecular bonding persists as far as 10*w* because of the solvophobic sequestration of water into nanostructured domains around choline. At 15*w*, we observed a step change in solvation where many of the DES structural motifs cease to be prevalent as water clusters become unfavorable. At this point, the system is best described as an aqueous solution of DES components at the molecular level. These developments give credence to the trend of researchers using hydration as a tool to overcome limitations of DESs, such as viscosity, and will aid the development of advanced DES and IL mixtures as greener processing and reaction media. DES compositions are highly variable, and whilst the nature of the transition highlighted by this work is likely to be similar to that in other systems, the water content at which this manifestation occurs may differ for systems with different compositions so that further case studies remain to be undertaken.

## Experimental Section

The pure DESs were prepared by mixing the components in the required molar ratio and heating at 60 °C until a homogenized, transparent phase had formed. Water was then added to meet the desired hydration level. The full set of DES compositions and isotopic substitutions are described in Supporting Information.

Samples were measured using the NIMROD or SANDALS total scattering instruments at the ISIS Neutron and Muon Source;^3^ raw diffraction data can be accessed using the ISIS‐ICAT system, under experiment numbers RB1510465, RB1610312, and RB1620479. The DES compositional purity (≤0.4 mol % absorbed atmospheric H_2_O) was assessed by contrasting the calculated and measured neutron differential scattering cross‐sections. The corrected diffraction data were analyzed using EPSR modeling;^16^, ^17^ corrected data is available via the University of Bath Research Data Archive system (DOI: https://doi.org/10.15125/BATH‐00359).

## Conflict of interest

The authors declare no conflict of interest.

## Supporting information

As a service to our authors and readers, this journal provides supporting information supplied by the authors. Such materials are peer reviewed and may be re‐organized for online delivery, but are not copy‐edited or typeset. Technical support issues arising from supporting information (other than missing files) should be addressed to the authors.

Supplementary
