# Supplementary material for: The Effect of Water upon Deep Eutectic Solvent Nanostructure: An Unusual Transition from Ionic Mixture to Aqueous Solution
Source: Angew Chem Int Ed Engl. 2017 May 29;56(33):9782–5. doi: 10.1002/anie.201702486 (PMC5596335; doi:10.1002/anie.201702486)
Supplement: Supplementary file 1 — Supplementary [file ANIE-56-9782-s001.pdf]

Angewandte Chemie
Eine Zeitschrift der Gesellschaft Deutscher Chemiker

Supporting Information

# The Effect of Water upon Deep Eutectic Solvent Nanostructure: An Unusual Transition from Ionic Mixture to Aqueous Solution

*Oliver S. Hammond, Daniel T. Bowron, and Karen J. Edler**

anie_201702486_sm_miscellaneous_information.pdf

# Supporting Information

## 1. Preparation of hydrated and isotopically-substituted reline samples

Choline chloride (≥99%) was obtained from Fisher, and urea (≥99%) was obtained from Sigma-Aldrich. $d_4$-urea ($CO(ND)_2$, 99.6% chemical purity, 99.8 atom-% D), and $d_9$-choline chloride ($(CD_3)_3N(CH_2)_2OHCl$, ≥99% chemical purity) were sourced from QMX laboratories. All chemicals were used as provided. Pure reline samples were first prepared by the literature route of mixing the two desired isotopes in the eutectic molar ratio, followed by heating at 60 ˚C with regular agitation. Water (Elga, 18.2 MΩ) or $D_2O$ (Sigma-Aldrich, ≥99.9% chemical purity and atom-% D) was then added to create a series of five choline chloride:urea:water isotope substitutions of H:H:H, H:D:D, D:H:D, D:D:H, and D:D:D. These isotope-substituted ternary DES-water mixtures were prepared in a series of DES:water molar ratios (defined as *w*), that are described in Table 1.

**Table 1.** The series of DES sample compositions that were used in these experiments.

| Mixture | ChCl:U:W ratio | Water / mol.% | [a]Water / wt.% |
|---|---|---|---|
| Reline-1*w* | 1:2:1 | 25.0 | 6.48 |
| Reline-2*w* | 1:2:2 | 40.0 | 12.18 |
| Reline-5*w* | 1:2:5 | 62.5 | 25.74 |
| Reline-10*w* | 1:2:10 | 76.9 | 40.95 |
| Reline-15*w* | 1:2:15 | 83.3 | 50.98 |
| Reline-20*w* | 1:2:20 | 87.0 | 58.10 |
| Reline-30*w* | 1:2:30 | 90.9 | 67.53 |

[a] The calculated weight percentage here is relevant only for ‘normal’ hydrogenous DES, and is a variable when the system is deuterium-substituted.

## 2. Neutron diffraction experiments

Neutron diffraction experiments were performed using different, yet largely analogous instruments. Samples of reline-1*w*, reline-2*w*, reline-15*w* and reline-20*w* were measured using the SANDALS diffractometer (beamtime allocations RB1510465 and RB1620479), located at TS1 of the STFC ISIS Neutron and Muon Source, Rutherford Appleton Laboratory, Harwell, UK. SANDALS is optimised for disordered light-element systems, using time-of-flight (TOF) neutrons with wavelength $0.05 \leq \lambda \leq 4.5$ Å, and a forward scattering detector geometry spanning an angular range of 3.8 - 35.4°, to give a *Q*-range of $0.1 \leq Q \leq 50$ Å$^{-1}$. The NIMROD diffractometer, located in TS2 of the same ISIS facility was also used (beamtime allocations RB1610312 and RB1620479) to measure reline-1*w*, reline-2*w*, reline-5*w*, reline-10*w*, and reline-30*w*. With a wide neutron TOF spectral range of $0.05 \leq \lambda \leq 11$ Å facilitated by the 100 ms pulse width of the 10 Hz TS2 source,[1] and detectors across the angular range 0.6 – 37.5°, NIMROD gives a wider *Q*-range than SANDALS. This wide *Q*-range of $0.01 \leq Q \leq 50$ Å$^{-1}$ facilitates measurement of larger structures, and would therefore allow the microscale phase separation suggested by D'Agostino et al. to be fully characterised.[2] When measurements were taken using both instruments, the results were functionally identical because no small-angle scattering signal was observed. For maximum consistency, SANDALS datasets were therefore used for EPSR modelling only when no NIMROD dataset was available (reline-15*w* and reline-20*w*), but instrumental similarity means that this has no bearing on the results.

For both instruments null-scattering, vacuum-sealed $Ti_{0.68}Zr_{0.32}$ sample cells with a path-length of 1 mm were filled with around 1.5 g of each sample, before being placed in a sample changer. The evacuated sample environment was regulated to 303 ± 0.1 K using a Julabo recirculating water/ethylene glycol temperature controller. Measurements were performed using a circularly-collimated neutron beam of 30 mm diameter, with a counting time of approximately 8 hours on SANDALS, or 2 hours on NIMROD. Empty sample cells, the empty instrument, and a 3 mm thick vanadium standard were also measured for data normalisation and instrument calibration. Data were processed using GudrunN software.[3] This involved adding corrections for attenuation, multiple scattering and the sample environment background, and normalising the data using the vanadium standard measurement. After a final correction for hydrogen inelasticity, a series of datasets are produced that can be analysed using Empirical Potential Structure Refinement (EPSR) modelling.

In each case, the accurate mass of each sample was recorded (weighing error ± 0.1 mg) in order to determine the sample purity. This was achieved by comparing the measured neutron scattering differential cross section (DCS) with the calculated DCS from the sample masses, using GudrunN neutron total scattering data reduction software.[3] Sample purity was determined to be adequate, as assessed by being within a neutron DCS error margin of ±2% for fully deuterated samples, and ±10% for fully hydrogenous samples. This is within the error of the diffraction experiment and sample preparation, with a higher error for more proton-rich samples because of the strong inelastic scattering of neutrons by hydrogen nuclei.[4] The experimental datasets are shown in Figure 1.

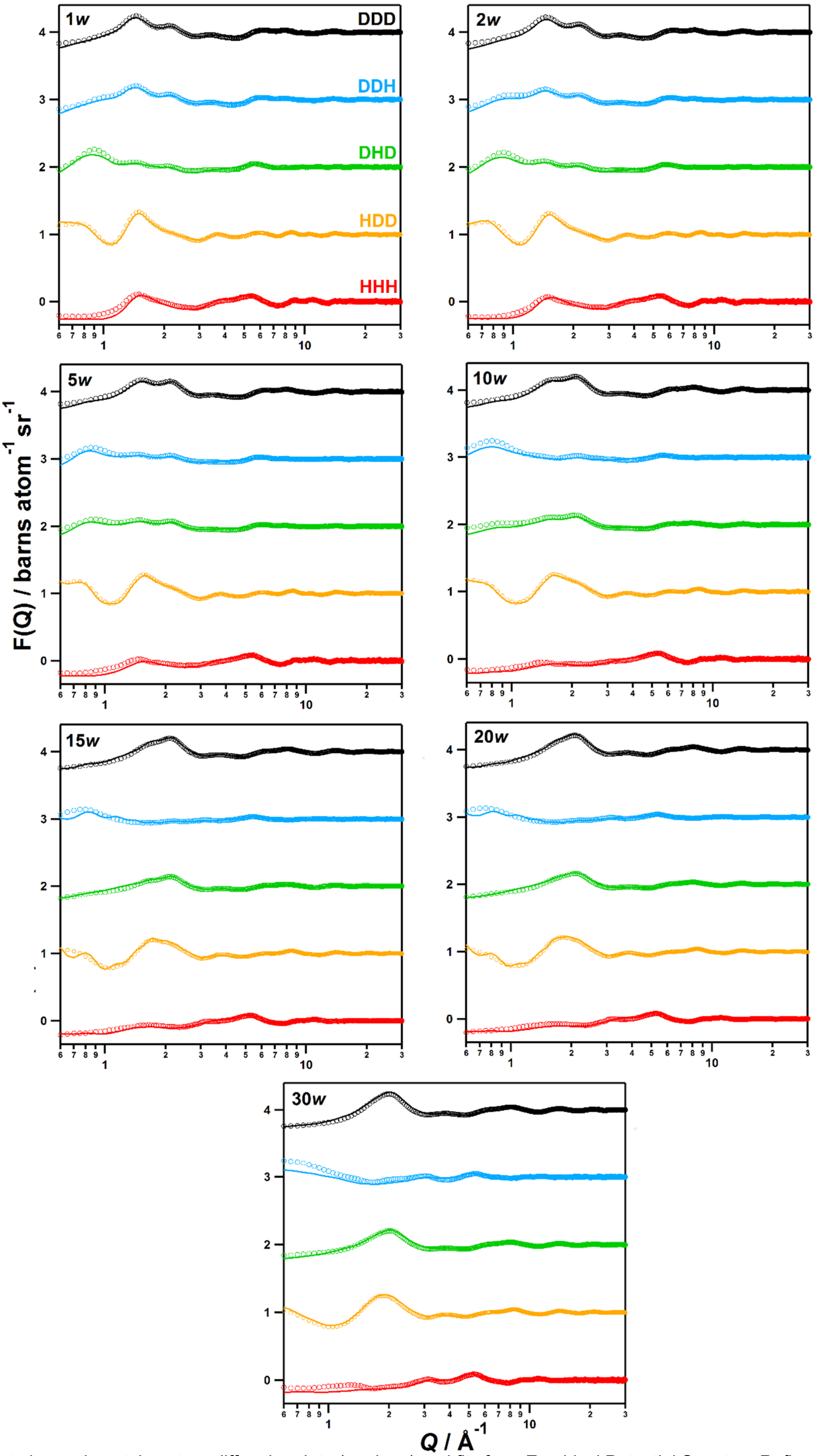


**Figure 1.** Corrected experimental neutron diffraction data (markers) and fits from Empirical Potential Structure Refinement atomistic models (solid lines) for the seven systems studied here.

## 3. Empirical Potential Structure Refinement (EPSR) modelling

Parameters for the water oxygen ‘$O_1$’ and hydrogen ‘$H_1$’ atoms were derived from the TIP3P model,[5] and are given in **Table 2**. The composition of each simulation box is shown in Table 3. The DES components of the hydrated EPSR models were parameterised and labelled in exactly the same way that was reported previously for the pure DES;[6] the atom labels are shown again for reference in Figure 2.

**Table 2.** EPSR parameters for water molecules that were used to model experimental diffraction data.

| Atom type | ε / kJ mol$^{-1}$ | σ / Å | q / e |
|---|---|---|---|
| $O_1$ | 0.634 | 3.151 | -0.834 |
| $H_1$ | 0.000 | 0.000 | 0.417 |

**Table 3.** The quantity of each molecule type used to build the simulation boxes is shown, alongside the total number of atoms in each box and the equilibrated box diameter. Atomic densities are calculated using a combination of literature values and experimental scattering data for H:H:H hydrated reline systems, and fall within the propagated errors from the literature measurements.[7]

| Mixture | Choline | Chloride | Urea | Water | Total | Box diameter / Å | Density / atoms Å$^{-3}$ |
|---|---|---|---|---|---|---|---|
| Reline-1*w* | 400 | 400 | 800 | 400 | 2000 | 54.025 | 0.104005 |
| Reline-2*w* | 300 | 300 | 600 | 600 | 1800 | 50.166 | 0.104553 |
| Reline-5*w* | 200 | 200 | 400 | 1000 | 1800 | 46.710 | 0.104012 |
| Reline-10*w* | 150 | 150 | 300 | 1500 | 2100 | 46.355 | 0.102405 |
| Reline-15*w* | 100 | 100 | 200 | 1500 | 1900 | 43.353 | 0.101861 |
| Reline-20*w* | 100 | 100 | 200 | 2000 | 2400 | 45.824 | 0.101846 |
| Reline-30*w* | 100 | 100 | 200 | 3000 | 3400 | 50.651 | 0.098500 |

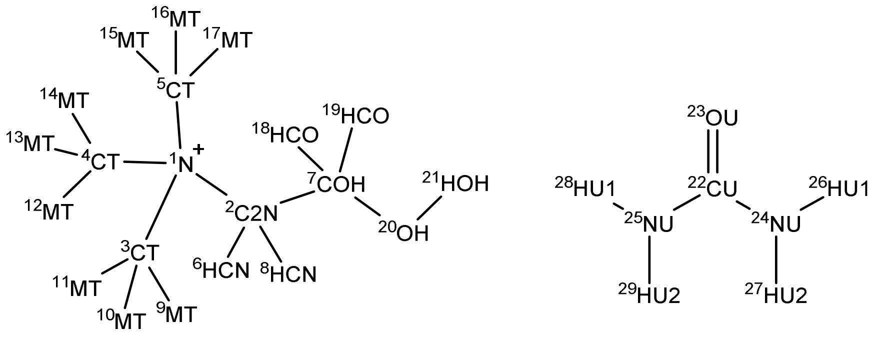


Figure 2. DES molecules used to create the EPSR reference potential. The shown atom type labels will be referred to in the text. Reprinted with permission from the Royal Society of Chemistry.[6]

Otherwise, the experimental procedure for the EPSR modelling was functionally identical to previous work on the pure reline DES.[6] The reference potential for each system was allowed to equilibrate to the experimental density described in Table 3, and the empirical potential was then allowed to equilibrate. Finally, ensemble information was interrogated from the model to gain information about the structure and bonding within these mixtures.

## 4. SDF plots

SDF plots are a way to aid with the visualisation of the 3D structure of a disordered liquid, with the surfaces showing the most likely places that a solvating species can be found around a certain molecule. Some additional spatial density function (SDF) plots are shown below, demonstrating some of the interesting and subtle changes occurring throughout the regime change. Firstly, the solvation of chloride by water is shown in supporting Figure 3, demonstrating the gradual increase in water-chloride interactions as the hydration level is increased. The appearance of the second solvation shell of chloride about water at 15*w* shows that water-chloride interactions are dominant over choline-chloride, and urea-chloride interactions. Secondly, the hydration of choline at 10*w*, the point at which this interaction is maximised, is shown in supporting Figure 4. Here, the water forms a radial solvation band around the choline, much like is seen in the pure solvent, for the choline-urea and choline-chloride interactions, but at closer length scale.

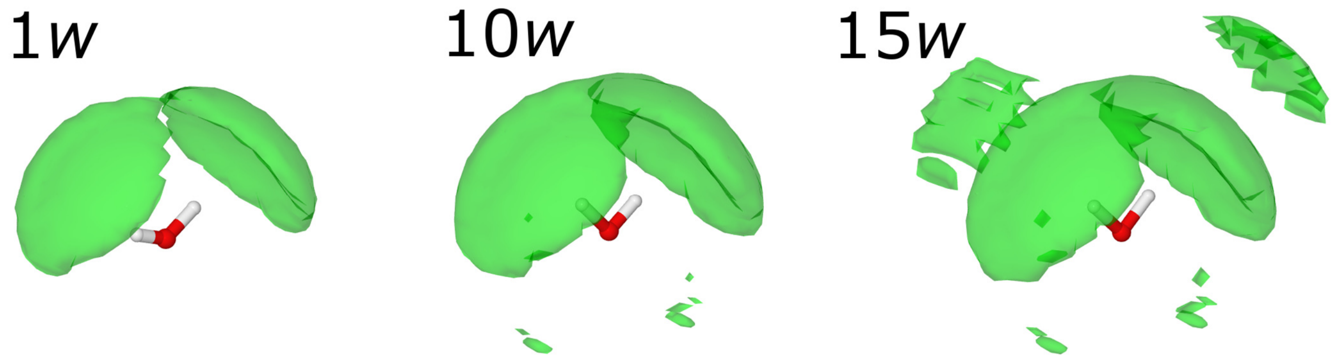


Figure 3. SDF plots centered on water molecules at different hydration levels, showing their solvation by chloride (green surfaces), and plotted at the 7.5% probability level. As the hydration level increases, the water-chloride interaction becomes more prevalent.

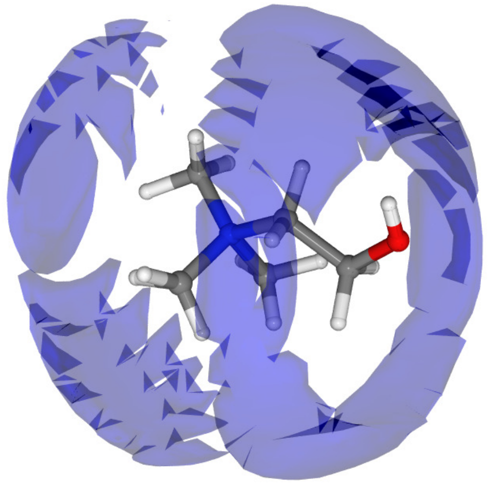

Figure 4. SDF plot showing hydration (blue surface) of choline at 10*w*, the point at which this interaction is maximised, and plotted at the 7.5% probability level.

## 5. Calculated Intermolecular Coordination Numbers

The tabulated intermolecular coordination numbers are shown in Tables 4 – 10, and these were calculated by integrating partial radial distribution functions (pRDFs) up to a radius $R_{max}$ corresponding with their first minima, where $R_{max}$ is accurate to a maximum of one data bin, *ie*. $R_{max}$ ± 0.02 $A^{-1}$. For these intermolecular coordination numbers the polyatomic molecular centres were defined as the $C_{2N}$ atom of choline, the $C_U$ atom of urea, and the $O_1$ atom of water.

**Table 4.** Calculated intermolecular coordination numbers for reline-1w. The calculated 'error' represents one standard deviation in coordination number.

| Molecule A | Molecule B | $R_{max}$ / Å | $N_{coord}$ | ± |
|---|---|---|---|---|
| Choline | Water | 6.2 | 2.50 | 1.56 |
| Urea | Water | 4.9 | 1.48 | 1.20 |
| Chloride | Water | 4.5 | 1.18 | 1.08 |
| Water | Water | 4.0 | 0.91 | 0.97 |
| Choline | Choline | 7.8 | 4.58 | 1.64 |
| Choline | Chloride | 6.4 | 3.19 | 1.12 |
| Choline | Chloride | 4.7 | 1.04 | 0.78 |
| Choline | Urea | 6.8 | 6.69 | 2.18 |
| Urea | Chloride | 5.2 | 1.76 | 0.94 |
| Urea | Urea | 5.7 | 4.01 | 1.71 |

**Table 5.** Calculated intermolecular coordination numbers for reline-2*w*. The calculated 'error' represents one standard deviation in coordination number.

| Molecule A | Molecule B | $R_{max}$ / Å | $N_{coord}$ | ± |
|---|---|---|---|---|
| Choline | Water | 6.2 | 4.81 | 2.22 |
| Urea | Water | 4.9 | 2.62 | 1.60 |
| Chloride | Water | 4.5 | 2.17 | 1.43 |
| Water | Water | 4.0 | 1.65 | 1.24 |
| Choline | Choline | 7.8 | 4.13 | 1.57 |
| Choline | Chloride | 6.4 | 3.01 | 1.14 |
| Choline | Chloride | 4.7 | 0.96 | 0.77 |
| Choline | Urea | 6.8 | 6.33 | 2.12 |
| Urea | Chloride | 5.2 | 1.60 | 0.92 |
| Urea | Urea | 5.7 | 3.67 | 1.62 |

**Table 6.** Calculated intermolecular coordination numbers for reline-5*w*. The calculated 'error' represents one standard deviation in coordination number.

| Molecule A | Molecule B | $R_{max}$ / Å | $N_{coord}$ | ± |
|---|---|---|---|---|
| Choline | Water | 6.2 | 10.08 | 3.03 |
| Urea | Water | 4.9 | 5.28 | 2.06 |
| Chloride | Water | 4.5 | 4.24 | 1.83 |
| Water | Water | 3.3 | 1.83 | 1.08 |
| Choline | Choline | 7.8 | 3.34 | 1.56 |
| Choline | Chloride | 6.4 | 2.46 | 1.08 |
| Choline | Chloride | 4.7 | 0.77 | 0.71 |
| Choline | Urea | 6.8 | 4.88 | 1.89 |
| Urea | Chloride | 5.2 | 1.26 | 0.86 |
| Urea | Urea | 5.7 | 3.09 | 1.58 |

**Table 7.** Calculated intermolecular coordination numbers for reline-10*w*. The calculated 'error' represents one standard deviation in coordination number.

| Molecule A | Molecule B | $R_{max}$ / Å | $N_{coord}$ | ± |
|---|---|---|---|---|
| Choline | Water | 6.2 | 15.37 | 3.08 |
| Urea | Water | 4.9 | 8.09 | 2.12 |
| Chloride | Water | 4.4 | 5.78 | 1.79 |
| Water | Water | 3.1 | 2.21 | 1.05 |
| Choline | Choline | 7.8 | 2.48 | 1.45 |
| Choline | Chloride | 6.4 | 1.87 | 1.03 |
| Choline | Chloride | 4.7 | 0.58 | 0.65 |
| Choline | Urea | 6.8 | 3.25 | 1.63 |
| Urea | Chloride | 5.2 | 0.90 | 0.78 |
| Urea | Urea | 5.7 | 2.39 | 1.36 |

**Table 8.** Calculated intermolecular coordination numbers for reline-15*w*. The calculated 'error' represents one standard deviation in coordination number.

| Molecule A | Molecule B | $R_{max}$ / Å | $N_{coord}$ | ± |
|---|---|---|---|---|
| Choline | Water | 6.2 | 10.15 | 6.32 |
| Urea | Water | 4.9 | 7.61 | 3.90 |
| Chloride | Water | 4.3 | 5.21 | 3.09 |
| Water | Water | 3.0 | 2.64 | 1.27 |
| Choline | Choline | 8.2 | 4.37 | 2.53 |
| Choline | Chloride | 6.4 | 1.89 | 1.03 |
| Choline | Chloride | 4.7 | 0.59 | 0.65 |
| Choline | Urea | 6.8 | 3.23 | 1.79 |
| Urea | Chloride | 5.2 | 0.84 | 0.77 |
| Urea | Urea | 5.7 | 2.27 | 1.46 |

**Table 9.** Calculated intermolecular coordination numbers for reline-20*w*. The calculated 'error' represents one standard deviation in coordination number.

| Molecule A | Molecule B | $R_{max}$ / Å | $N_{coord}$ | ± |
|---|---|---|---|---|
| Choline | Water | 6.2 | 14.18 | 5.44 |
| Urea | Water | 4.9 | 9.19 | 3.30 |
| Chloride | Water | 4.3 | 6.29 | 2.73 |
| Water | Water | 3.0 | 2.63 | 1.18 |
| Choline | Choline | 8.2 | 3.37 | 1.84 |
| Choline | Chloride | 6.4 | 1.50 | 0.99 |
| Choline | Chloride | 4.7 | 0.48 | 0.61 |
| Choline | Urea | 6.8 | 2.64 | 1.54 |
| Urea | Chloride | 5.2 | 0.67 | 0.69 |
| Urea | Urea | 5.7 | 1.78 | 1.27 |

**Table 10.** Calculated intermolecular coordination numbers for reline-30*w*. The calculated 'error' represents one standard deviation in coordination number.

| Molecule A | Molecule B | $R_{max}$ / Å | $N_{coord}$ | ± |
|---|---|---|---|---|
| Choline | Water | 6.2 | 19.03 | 4.07 |
| Urea | Water | 4.9 | 10.84 | 2.50 |
| Chloride | Water | 4.2 | 6.69 | 1.91 |
| Water | Water | 3.0 | 2.68 | 1.09 |
| Choline | Choline | 8.2 | 2.05 | 1.48 |
| Choline | Chloride | 6.4 | 1.01 | 0.86 |
| Choline | Chloride | 4.7 | 0.32 | 0.51 |
| Choline | Urea | 6.8 | 1.56 | 1.17 |
| Urea | Chloride | 5.2 | 0.48 | 0.62 |
| Urea | Urea | 5.7 | 1.18 | 1.02 |

## 6. Integrated partial (site-site) coordination numbers

As with the intermolecular coordination numbers, the tabulated site-site radial distribution function analysis is shown in Tables 11 – 17. These values were calculated by integrating a selection of partial radial distribution functions (pRDFs) best describing the specific intermolecular bonding, up to a radius $R_{max}$ corresponding with their first minima, where $R_{max}$ is accurate to a maximum of one data bin, *ie*. $R_{max}$ ± 0.02 $A^{-1}$. For these coordination numbers, the 'error' reflects the disorder present in the liquid, rather than a lack of general confidence in the data. In this case, 'important' coordination numbers are those which have a variance significantly smaller than the coordination number itself, signifying a persistent correlation.

**Table 11.** Site-site coordination numbers calculated from selected pRDFs of reline-1*w*. The calculated 'error' represents one standard deviation in coordination number.

| Atom A | Atom B | $R_{max}$ / Å | $N_{coord}$ | ± |
|---|---|---|---|---|
| Cl | $C_U$ | 5.0 | 3.25 | 1.49 |
| Cl | $N_U$ | 3.9 | 3.10 | 1.67 |
| Cl | $H_{U1}$ | 3.0 | 1.52 | 1.36 |
| Cl | $H_{U2}$ | 3.0 | 1.02 | 0.93 |
| $H_{OH}$ | Cl | 3.2 | 0.55 | 0.51 |
| $M_T$ | Cl | 4.0 | 0.59 | 0.62 |
| $H_{CN}$ | Cl | 4.0 | 0.59 | 0.61 |
| $H_{CO}$ | Cl | 4.0 | 0.59 | 0.62 |
| $H_{CN}$ | $O_U$ | 3.5 | 0.48 | 0.62 |
| $M_T$ | $O_U$ | 3.4 | 0.52 | 0.65 |
| $H_{CO}$ | $O_U$ | 3.6 | 0.54 | 0.67 |
| $H_{OH}$ | $O_U$ | 2.5 | 0.19 | 0.40 |
| $H_{OH}$ | $N_U$ | 4.3 | 2.49 | 1.60 |
| N | N | 6.8 | 2.61 | 1.28 |
| N | $C_{2N}$ | 7.5 | 4.98 | 1.58 |
| N | $C_{OH}$ | 6.2 | 2.88 | 1.24 |
| N | $O_H$ | 5.2 | 2.08 | 0.98 |
| N | $H_{OH}$ | 5.5 | 2.36 | 1.10 |
| $O_U$ | $N_U$ | 3.5 | 3.31 | 1.05 |
| $O_U$ | $H_{U1}$ | 2.6 | 0.46 | 0.73 |
| $O_U$ | $H_{U2}$ | 2.6 | 1.73 | 0.87 |
| $N_U$ | $H_{U1}$ | 4.6 | 4.87 | 2.01 |
| $N_U$ | $H_{U2}$ | 4.6 | 4.97 | 1.78 |
| Cl | Cl | 6.0 | 1.23 | 0.91 |
| N | $O_1$ | 5.8 | 2.09 | 1.41 |
| $H_{OH}$ | $O_1$ | 2.3 | 0.07 | 0.26 |
| $H_{CN}$ | $O_1$ | 3.5 | 0.28 | 0.52 |
| $H_{CO}$ | $O_1$ | 3.6 | 0.32 | 0.56 |
| Cl | $O_1$ | 4.3 | 1.09 | 1.03 |
| $O_1$ | $O_1$ | 4.2 | 0.99 | 1.02 |

**Table 12.** Site-site coordination numbers calculated from selected pRDFs of reline-2*w*. The calculated 'error' represents one standard deviation in coordination number.

| Atom A | Atom B | $R_{max}$ / Å | $N_{coord}$ | ± |
|---|---|---|---|---|
| Cl | $C_U$ | 5.0 | 2.93 | 1.40 |
| Cl | $N_U$ | 3.9 | 2.77 | 1.59 |
| Cl | $H_{U1}$ | 3.0 | 1.38 | 1.32 |
| Cl | $H_{U2}$ | 3.0 | 0.89 | 0.86 |
| $H_{OH}$ | Cl | 3.2 | 0.53 | 0.51 |
| $M_T$ | Cl | 4.0 | 0.54 | 0.61 |
| $H_{CN}$ | Cl | 4.0 | 0.55 | 0.60 |
| $H_{CO}$ | Cl | 4.0 | 0.54 | 0.61 |
| $H_{CN}$ | $O_U$ | 3.5 | 0.45 | 0.61 |
| $M_T$ | $O_U$ | 3.4 | 0.48 | 0.63 |
| $H_{CO}$ | $O_U$ | 3.6 | 0.50 | 0.64 |
| $H_{OH}$ | $O_U$ | 2.5 | 0.17 | 0.38 |
| $H_{OH}$ | $N_U$ | 4.3 | 2.34 | 1.55 |
| N | N | 6.8 | 2.27 | 1.23 |
| N | $C_{2N}$ | 7.5 | 4.53 | 1.50 |
| N | $C_{OH}$ | 6.2 | 2.66 | 1.16 |
| N | $O_H$ | 5.2 | 1.96 | 0.92 |
| N | $H_{OH}$ | 5.6 | 2.29 | 1.06 |
| $O_U$ | $N_U$ | 3.5 | 3.19 | 1.01 |
| $O_U$ | $H_{U1}$ | 2.6 | 0.42 | 0.70 |
| $O_U$ | $H_{U2}$ | 2.6 | 1.72 | 0.85 |
| $N_U$ | $H_{U1}$ | 4.6 | 4.58 | 1.92 |
| $N_U$ | $H_{U2}$ | 4.6 | 4.69 | 1.71 |
| Cl | Cl | 6.0 | 1.14 | 0.90 |
| N | $O_1$ | 5.6 | 3.65 | 1.90 |
| $H_{OH}$ | $O_1$ | 2.3 | 0.14 | 0.35 |
| $H_{CN}$ | $O_1$ | 3.5 | 0.53 | 0.70 |
| $H_{CO}$ | $O_1$ | 3.6 | 0.62 | 0.77 |
| Cl | $O_1$ | 4.3 | 1.99 | 1.36 |
| $O_1$ | $O_1$ | 4.0 | 1.65 | 1.24 |

**Table 13.** Site-site coordination numbers calculated from selected pRDFs of reline-5*w*. The calculated 'error' represents one standard deviation in coordination number.

| Atom A | Atom B | $R_{max}$ / Å | $N_{coord}$ | ± |
|---|---|---|---|---|
| Cl | $C_U$ | 5.1 | 2.40 | 1.35 |
| Cl | $N_U$ | 4.0 | 2.28 | 1.53 |
| Cl | $H_{U1}$ | 3.0 | 1.08 | 1.17 |
| Cl | $H_{U2}$ | 3.0 | 0.65 | 0.77 |
| $H_{OH}$ | Cl | 3.2 | 0.43 | 0.50 |
| $M_T$ | Cl | 4.0 | 0.42 | 0.55 |
| $H_{CN}$ | Cl | 4.0 | 0.44 | 0.56 |
| $H_{CO}$ | Cl | 4.0 | 0.43 | 0.56 |
| $H_{CN}$ | $O_U$ | 3.5 | 0.33 | 0.54 |
| $M_T$ | $O_U$ | 3.4 | 0.36 | 0.55 |
| $H_{CO}$ | $O_U$ | 3.6 | 0.37 | 0.56 |
| $H_{OH}$ | $O_U$ | 2.5 | 0.13 | 0.35 |
| $H_{OH}$ | $N_U$ | 4.3 | 1.78 | 1.42 |
| N | N | 6.8 | 1.76 | 1.16 |
| N | $C_{2N}$ | 7.5 | 3.85 | 1.49 |
| N | $C_{OH}$ | 6.2 | 2.32 | 1.11 |
| N | $O_H$ | 5.3 | 1.81 | 0.88 |
| N | $H_{OH}$ | 5.6 | 2.01 | 0.98 |
| $O_U$ | $N_U$ | 3.5 | 2.96 | 0.96 |
| $O_U$ | $H_{U1}$ | 2.6 | 0.33 | 0.64 |
| $O_U$ | $H_{U2}$ | 2.6 | 1.58 | 0.83 |
| $N_U$ | $H_{U1}$ | 4.6 | 4.13 | 1.83 |
| $N_U$ | $H_{U2}$ | 4.6 | 4.19 | 1.65 |
| Cl | Cl | 6.0 | 0.85 | 0.80 |
| N | $O_1$ | 5.4 | 6.82 | 2.41 |
| $H_{OH}$ | $O_1$ | 2.3 | 0.25 | 0.44 |
| $H_{CN}$ | $O_1$ | 3.6 | 1.13 | 0.95 |
| $H_{CO}$ | $O_1$ | 3.6 | 1.23 | 1.01 |
| Cl | $O_1$ | 4.3 | 3.83 | 1.71 |
| $O_1$ | $O_1$ | 3.5 | 2.07 | 1.16 |

**Table 14.** Site-site coordination numbers calculated from selected pRDFs of reline-10*w*. The calculated 'error' represents one standard deviation in coordination number.

| Atom A | Atom B | $R_{max}$ / Å | $N_{coord}$ | ± |
|---|---|---|---|---|
| Cl | $C_U$ | 5.2 | 1.79 | 1.24 |
| Cl | $N_U$ | 4.1 | 1.71 | 1.39 |
| Cl | $H_{U1}$ | 3.0 | 0.76 | 1.00 |
| Cl | $H_{U2}$ | 3.0 | 0.44 | 0.65 |
| $H_{OH}$ | Cl | 3.2 | 0.35 | 0.48 |
| $M_T$ | Cl | 4.0 | 0.30 | 0.49 |
| $H_{CN}$ | Cl | 4.0 | 0.33 | 0.51 |
| $H_{CO}$ | Cl | 4.0 | 0.32 | 0.51 |
| $H_{CN}$ | $O_U$ | 3.5 | 0.22 | 0.45 |
| $M_T$ | $O_U$ | 3.5 | 0.25 | 0.47 |
| $H_{CO}$ | $O_U$ | 3.6 | 0.27 | 0.49 |
| $H_{OH}$ | $O_U$ | 2.5 | 0.10 | 0.30 |
| $H_{OH}$ | $N_U$ | 4.3 | 1.21 | 1.19 |
| N | N | 7.3 | 1.88 | 1.25 |
| N | $C_{2N}$ | 7.7 | 3.41 | 1.43 |
| N | $C_{OH}$ | 6.4 | 2.09 | 1.01 |
| N | $O_H$ | 5.4 | 1.62 | 0.77 |
| N | $H_{OH}$ | 5.8 | 1.84 | 0.90 |
| $O_U$ | $N_U$ | 3.6 | 2.78 | 0.89 |
| $O_U$ | $H_{U1}$ | 2.6 | 0.25 | 0.55 |
| $O_U$ | $H_{U2}$ | 2.6 | 1.51 | 0.77 |
| $N_U$ | $H_{U1}$ | 4.6 | 3.49 | 1.60 |
| $N_U$ | $H_{U2}$ | 4.6 | 3.57 | 1.45 |
| Cl | Cl | 6.0 | 0.58 | 0.69 |
| N | $O_1$ | 5.4 | 10.12 | 2.42 |
| $H_{OH}$ | $O_1$ | 2.3 | 0.35 | 0.48 |
| $H_{CN}$ | $O_1$ | 3.6 | 1.61 | 1.05 |
| $H_{CO}$ | $O_1$ | 3.6 | 1.75 | 1.12 |
| Cl | $O_1$ | 4.2 | 5.12 | 1.66 |
| $O_1$ | $O_1$ | 3.4 | 2.70 | 1.16 |

**Table 15.** Site-site coordination numbers calculated from selected pRDFs of reline-15*w*. The calculated 'error' represents one standard deviation in coordination number.

| Atom A | Atom B | $R_{max}$ / Å | $N_{coord}$ | ± |
|---|---|---|---|---|
| Cl | $C_U$ | 5.2 | 1.67 | 1.25 |
| Cl | $N_U$ | 4.2 | 1.75 | 1.50 |
| Cl | $H_{U1}$ | 3.0 | 0.77 | 1.05 |
| Cl | $H_{U2}$ | 3.0 | 0.43 | 0.65 |
| $H_{OH}$ | Cl | 3.2 | 0.38 | 0.49 |
| $M_T$ | Cl | 4.0 | 0.32 | 0.50 |
| $H_{CN}$ | Cl | 4.1 | 0.36 | 0.52 |
| $H_{CO}$ | Cl | 4.1 | 0.37 | 0.53 |
| $H_{CN}$ | $O_U$ | 3.6 | 0.26 | 0.49 |
| $M_T$ | $O_U$ | 3.5 | 0.26 | 0.49 |
| $H_{CO}$ | $O_U$ | 3.6 | 0.27 | 0.50 |
| $H_{OH}$ | $O_U$ | 2.5 | 0.12 | 0.33 |
| $H_{OH}$ | $N_U$ | 4.3 | 1.28 | 1.26 |
| N | N | 7.7 | 3.58 | 2.22 |
| N | $C_{2N}$ | 8.0 | 5.10 | 2.43 |
| N | $C_{OH}$ | 6.7 | 3.06 | 1.56 |
| N | $O_H$ | 5.5 | 2.05 | 1.07 |
| N | $H_{OH}$ | 6.0 | 2.47 | 1.32 |
| $O_U$ | $N_U$ | 3.8 | 2.94 | 1.03 |
| $O_U$ | $H_{U1}$ | 2.6 | 0.26 | 0.57 |
| $O_U$ | $H_{U2}$ | 2.6 | 1.52 | 0.78 |
| $N_U$ | $H_{U1}$ | 4.6 | 3.44 | 1.69 |
| $N_U$ | $H_{U2}$ | 4.6 | 3.46 | 1.52 |
| Cl | Cl | 6.0 | 0.48 | 0.63 |
| N | $O_1$ | 5.5 | 7.00 | 4.49 |
| $H_{OH}$ | $O_1$ | 2.3 | 0.25 | 0.44 |
| $H_{CN}$ | $O_1$ | 3.6 | 0.99 | 1.08 |
| $H_{CO}$ | $O_1$ | 3.6 | 1.09 | 1.17 |
| Cl | $O_1$ | 4.1 | 4.59 | 2.73 |
| $O_1$ | $O_1$ | 3.3 | 3.59 | 1.56 |

**Table 16.** Site-site coordination numbers calculated from selected pRDFs of reline-20*w*. The calculated 'error' represents one standard deviation in coordination number.

| Atom A | Atom B | $R_{max}$ / Å | $N_{coord}$ | ± |
|---|---|---|---|---|
| Cl | $C_U$ | 5.3 | 1.39 | 1.14 |
| Cl | $N_U$ | 4.2 | 1.39 | 1.32 |
| Cl | $H_{U1}$ | 3.0 | 0.62 | 0.91 |
| Cl | $H_{U2}$ | 3.0 | 0.33 | 0.56 |
| $H_{OH}$ | Cl | 3.2 | 0.29 | 0.46 |
| $M_T$ | Cl | 4.0 | 0.25 | 0.46 |
| $H_{CN}$ | Cl | 4.1 | 0.29 | 0.48 |
| $H_{CO}$ | Cl | 4.1 | 0.28 | 0.48 |
| $H_{CN}$ | $O_U$ | 3.6 | 0.21 | 0.44 |
| $M_T$ | $O_U$ | 3.6 | 0.23 | 0.46 |
| $H_{CO}$ | $O_U$ | 3.6 | 0.22 | 0.45 |
| $H_{OH}$ | $O_U$ | 2.6 | 0.11 | 0.32 |
| $H_{OH}$ | $N_U$ | 4.5 | 1.28 | 1.29 |
| N | N | 8.0 | 3.10 | 1.74 |
| N | $C_{2N}$ | 8.3 | 4.48 | 1.85 |
| N | $C_{OH}$ | 6.8 | 2.69 | 1.29 |
| N | $O_H$ | 5.6 | 1.90 | 0.94 |
| N | $H_{OH}$ | 6.2 | 2.30 | 1.14 |
| $O_U$ | $N_U$ | 3.9 | 2.80 | 0.95 |
| $O_U$ | $H_{U1}$ | 2.6 | 0.20 | 0.50 |
| $O_U$ | $H_{U2}$ | 2.6 | 1.46 | 0.75 |
| $N_U$ | $H_{U1}$ | 4.6 | 3.02 | 1.47 |
| $N_U$ | $H_{U2}$ | 4.6 | 3.04 | 1.37 |
| Cl | Cl | 6.0 | 0.36 | 0.57 |
| N | $O_1$ | 5.6 | 10.29 | 4.11 |
| $H_{OH}$ | $O_1$ | 2.3 | 0.34 | 0.48 |
| $H_{CN}$ | $O_1$ | 3.6 | 1.35 | 1.14 |
| $H_{CO}$ | $O_1$ | 3.6 | 1.50 | 1.22 |
| Cl | $O_1$ | 4.1 | 5.51 | 2.41 |
| $O_1$ | $O_1$ | 3.3 | 3.56 | 1.40 |

**Table 17.** Site-site coordination numbers calculated from selected pRDFs of reline-30*w*. The calculated 'error' represents one standard deviation in coordination number.

| Atom A | Atom B | $R_{max}$ / Å | $N_{coord}$ | ± |
|---|---|---|---|---|
| Cl | $C_U$ | 5.4 | 1.05 | 0.96 |
| Cl | $N_U$ | 4.4 | 1.12 | 1.19 |
| Cl | $H_{U1}$ | 3.1 | 0.49 | 0.83 |
| Cl | $H_{U2}$ | 3.1 | 0.25 | 0.49 |
| $H_{OH}$ | Cl | 3.2 | 0.21 | 0.40 |
| $M_T$ | Cl | 4.0 | 0.17 | 0.38 |
| $H_{CN}$ | Cl | 4.1 | 0.20 | 0.42 |
| $H_{CO}$ | Cl | 4.2 | 0.20 | 0.41 |
| $H_{CN}$ | $O_U$ | 3.6 | 0.13 | 0.35 |
| $M_T$ | $O_U$ | 3.6 | 0.14 | 0.37 |
| $H_{CO}$ | $O_U$ | 3.6 | 0.12 | 0.34 |
| $H_{OH}$ | $O_U$ | 2.6 | 0.06 | 0.23 |
| $H_{OH}$ | $N_U$ | 4.4 | 0.66 | 0.93 |
| N | N | 8.2 | 2.04 | 1.43 |
| N | $C_{2N}$ | 8.6 | 3.32 | 1.56 |
| N | $C_{OH}$ | 6.8 | 2.04 | 1.09 |
| N | $O_H$ | 5.6 | 1.56 | 0.78 |
| N | $H_{OH}$ | 6.3 | 1.82 | 0.95 |
| $O_U$ | $N_U$ | 3.9 | 2.52 | 0.78 |
| $O_U$ | $H_{U1}$ | 2.6 | 0.13 | 0.40 |
| $O_U$ | $H_{U2}$ | 2.6 | 1.35 | 0.70 |
| $N_U$ | $H_{U1}$ | 4.6 | 2.48 | 1.23 |
| $N_U$ | $H_{U2}$ | 4.6 | 2.53 | 1.13 |
| Cl | Cl | 6.0 | 0.25 | 0.48 |
| N | $O_1$ | 6.0 | 17.01 | 3.63 |
| $H_{OH}$ | $O_1$ | 2.3 | 0.47 | 0.51 |
| $H_{CN}$ | $O_1$ | 3.6 | 1.80 | 1.14 |
| $H_{CO}$ | $O_1$ | 3.6 | 1.97 | 1.22 |
| Cl | $O_1$ | 4.1 | 6.24 | 1.82 |
| $O_1$ | $O_1$ | 3.3 | 3.52 | 1.22 |

## 7. References


[1] D. T. Bowron, A. K. Soper, K. Jones, S. Ansell, S. Birch, J. Norris, L. Perrott, D. Riedel, N. J. Rhodes, S. R. Wakefield, et al., *Rev. Sci. Instrum.* **2010**, *81*, 033905.

[2] C. D'Agostino, L. F. Gladden, M. D. Mantle, A. P. Abbott, I. Ahmed, Essa, A. Y. M. Al-Murshedi, R. C. Harris, *Phys. Chem. Chem. Phys.* **2015**, 15297–15304.

[3] A. K. Soper, *GudrunN and GudrunX: Programs for Correcting Raw Neutron and X-Ray Diffraction Data to Differential Scattering Cross Section. Rutherford Appleton Laboratory Technical Report RAL-TR-2011-013*, **2011**.

[4] A. K. Soper, *Mol. Phys.* **2009**, *107*, 1667–1684.

[5] P. Mark, L. Nilsson, *J. Phys. Chem. A* **2001**, *105*, 9954–9960.

[6] O. S. Hammond, D. T. Bowron, K. J. Edler, *Green Chem.* **2016**, *18*, 2736–2744.

[7] A. . Yadav, S. Pandey, *J. Chem. Eng. Data.* **2014**, 2221-2229.
